# Usefulness of grayscale inverted images in addition to standard images in digital mammography

**DOI:** 10.1186/s12880-017-0196-6

**Published:** 2017-04-18

**Authors:** Ayşegül Altunkeser, M. Kazım Körez

**Affiliations:** 1 Department of Radiology, Konya Education and Research Hospital, Konya, Turkey; 2 Department of Statistics, Faculty of Science, Selcuk University, Konya, Turkey; 3 Konya Eğitim ve Araştırma Hastanesi, Radyoloji Bölümü, Hacı Şaban Mah, Meram Yeni Yol Caddesi, No: 97, PK: 42090 Meram, Konya Turkey

**Keywords:** Digital mammography, Macrocalcification, Microcalcification, Grayscale inverted images

## Abstract

**Background:**

Mammography is essential for early diagnosis of breast cancer, which is the most common type of cancer in females that is associated with a high mortality rate. We investigated whether evaluation of the grayscale inverted images of mammograms would aid in increasing the diagnostic sensitivity of the mammographic imaging technique.

**Methods:**

Our study included 636 mammograms of 159 women who had undergone digital mammography. Standard, grayscale inverted, and standard plus grayscale inverted images were sequentially examined three times, at 15-day intervals, for the presence or assessment of pathological changes in the skin, calcification, asymmetric density, mass lesions, structural distortions, and intramammary and axillary lymph nodes. To determine whether grayscale inverted image assessment improved detection rates, the results of the three assessment modes were compared using Cochran’s Q test and the McNemar test (*p* < 0.05 was considered statistically significant).

**Results:**

The average age of 159 patients was 50.4 years (range, 35–80 years). There were significant differences among the three assessment modes with respect to calcification and intramammary lymph nodes (*p* < 0.05); however, no significant differences were observed for the detection of other parameters.

**Conclusions:**

Assessment of grayscale inverted images in addition to standard images facilitates the detection of microcalcification.

## Background

Breast cancer is the most frequent women’s cancer, with high mortality rates in Turkey and other parts of the world. In Turkey, its incidence is 40.6 per 100,000 women (http://www.kanser.gov.tr/Dosya/ca_istatistik/2009kanseraporu). Early diagnosis can decrease the likelihood of mortality and requires scanning. It has been reported that scanning of women over 50 years of age reduces breast cancer-related mortality rates by 3–36% [1, 2].

Mammography is the most effective screening method for breast cancer [3]. However, mammographic assessment is difficult and requires experience owing to the complex structure of mammary tissue. The false negative range in mammographic detection of breast cancer is 10–25%. Dense mammary structure, inferior mammogram quality, overlooked details of early breast cancers, and fatigue or carelessness of the radiologist may account for the high percentage of false negatives [4]. To lower this percentage, a double-reading system is recommended [5] but is costly and requires two professional readers and is thus impractical. Computer-aided detection (CAD) is an alternative method, especially in busy crowded centers that focus on mammography [6].

Unfortunately, not all mammography centers facilitate double reading or CAD system. In our center, one radiologist assesses the data obtained via digital mammography without CAD. In view of the fact that digital mammography is a digitalized system, digitalization allows different representations of the images. Therefore, in this paper we studied if the diagnostic assessment of grayscale inverted mammograms improved compared to a standard representation of mammographic images when double-reading or CAD is impossible.

## Methods

Our study included 636 mammograms of 159 women who had undergone mammography at Dr. Faruk Sükan Obstetrics and Pediatrics Hospital between January 5 and February 4, 2015. Scanning was performed for diagnostic purposes, in the mediolateral oblique and cranio-caudal views, and using a digital mammography device, which has ring shaped gantry, 24X30 cm Amorphous Selenium detector, Tungsten anode tube (Giotto Image 3D, IMS, Bologna, Italy). This study was approved by the ethics committee of Necmettin Erbakan University. Mammograms were evaluated by an experienced radiologist. Mammography was performed according to the Breast Imaging Reporting and Data System (BIRADS) as recommended by the American College of Radiology [7].

Initially, the parenchymal patterns on mammograms were evaluated. and categorized as follows: type A, the whole breast consists predominantly of fat tissue; type B, the whole breast contains fibroglandular densities as well as fat tissue; type C, the density of the breast parenchyma is heterogeneous; and type D, the breast parenchyma is highly dense [7]. Pathological changes in the skin (increased thickness, mass, or shrinkage), calcification, asymmetric density, mass lesion, structural distortion, and intramammary and axillary lymph nodes were then assessed and scored as present or absent. Calcifications were classified as macro or micro (<0.5 mm) regardless of their morphological properties and distribution patterns. Asymmetric density included both focal and regional asymmetric density detected in either or both views. Densities with a three-dimensional structure and observed in both views were defined as mass. Structural distortion was defined as shrinkage withdrawal without mass. Centrically radiolucent lymph nodes with regular margins and oval-shaped nodular opacities were classified as intramammary (present in the breast parenchyma) or axillary (present in the axillary region).

**Fig. 1:**
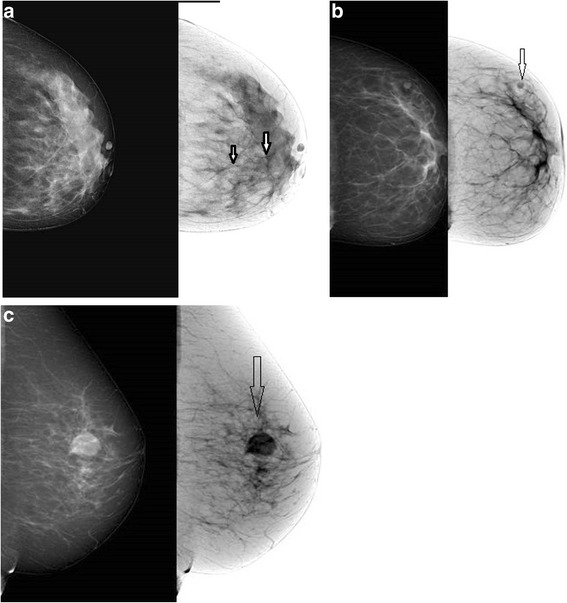
**a**-**c** Standard and grayscale inverted image. *Arrows* indicate the early vascular macrocalcification, intramammary lymph node and nodular density with smooth contour respectively

Initial assessments were performed on standard images (the std method). Results were reported as present or absent or scored by using the BIRADS. In the final BIRADS assessment, BIRADS scores were defined as follows: 0, more examination needed; 1, normal; 2, benign; 3, probably benign; 4, suspicious; 5, highly suspicious; and 6, malignant [7]. At least 15 days after the first assessment, the same mammograms were evaluated as above using only the grayscale inverted images (the gsi method). At least 15 days later, a third assessment was performed using both standard and grayscale inverted images (the std + gsi method) (Fig. 1a-c). Cochran’s Q test was used to compare the results obtained with the three types of image evaluation. The McNemar multiple comparison test was used to compare the different methods (*p* < 0.05 was considered statistically significant). The intraobserver concordance among three assessment methods was measured by using the Fleiss’ Kappa test.

## Results

The average age of 159 patients in our study was 50.4 years (range, 35–80 years; standard deviation, 7.81 years). The parenchymal patterns on standard images were as follows: type A, 6 (3.8%) women; type B, 68 (42.8%) women; type C, 54 (34%) women; and type D, 31 (19.5%) women. According to the BIRADS, 45 (28.3%) mammograms were categorized as 0, 39 (24.5%) as 1, 74 (46.5%) as 2, and 1 (0.6%) as 3 (Table 1). There were no cases with BIRADS 4,5 and 6 score.

**Table 1:** Parenchyma pattern of mammograms and BIRADS classification

Pattern	A	B	C	D				Total
Standard İmage	6 (3.8%)	68 (42.8%)	54 (34%)	31 (19.5%)				159 (100%)
BIRADS	0	1	2	3	4	5	6	Total
Standard İmage	45 (28.3%)	39 (24.5%)	74 (46.5%)	1 (0.6%)	0	0	0	159 (100%)

*BIRADS* Breast Imaging Reporting and Data System

The intraobserver concordance for the results of the three assessment methods (standard, grayscale inverted, and std + gsi) is shown in Table 2. There were statistically significant differences in presence/absence of macrocalcification, microcalcification, and intramammary lymph nodes among the methods (*p* < 0.05) (Table 3), but not in the other parameters (Table 4).

**Table 2:** Intraobserver concordance assessment

Parameter	Fleiss Kappa	Concordance
Skin Pathology	0.908	Perfect Concordance
Macrocalcification	0.916	Perfect Concordance
Microcalcification	0.597	Moderate Concordance
Asymmetric Density	0.646	High Concordance
Structural Distortion	0.662	High Concordance
Mass	0.703	High Concordance
İntramammary Lymph Nodes	0.809	High Concordance
Axillary Lymph Nodes	0.905	Perfect Concordance

**Table 3:** Significant differences between the image types

Parameter	Image type	No (%)	Yes (%)	Cochran’s Q	*p*	Multiple comparison
Macro-calcification	(a) Standard	234 (73.58)	84 (26.42)	8.375	0.015*	(c)
	(b) Grayscale inverted	232 (72.96)	86 (27.04)			
	(c) Std + Gsi	225 (70.75)	93 (29.25)			(a)
Micro-calcification	(a) Standard	287 (90.25)	31 (9.75)	68.509	<0.001¶	(b) (c)
	(b) Grayscale inverted	265 (83.33)	53 (16.67)			(a) (c)
	(c) Std + Gsi	237 (74.53)	81 (25.47)			(a) (b)
Intramammary Lymph Nodes	(a) Standard	269 (84.59)	49 (15.41)	6.461	0.039†	(c)
	(b) Grayscale inverted	261 (82.08)	57 (17.92)			
	(c) Std + Gsi	259 (81.45)	59 (18.55)			(a)

*Std + Gsi* standard plus grayscale inverted image. *: a versus c. ¶: a versus b, a versus c, and b versus c. †: a versus c

**Table 4:** Insignificant differences between the image types

Parameter	Image	No (%)	Yes (%)	Cochran’s Q	*p*
Skin Pathology	Standard	314 (98.75)	4 (1.25)	2.000	0.367
	Grayscale inverted	315 (99.05)	3 (0.95)		
	Std + Gsi	314 (98.75)	4 (1.25)		
Asymmetric Density	Standard	288 (90.57)	30 (9.43)	0.266	0.875
	Grayscale inverted	286 (89.94)	32 (10.06)		
	Std + Gsi	286 (89.94)	32 (10.06)		
Structural Distortion	Standard	315 (99.06)	3 (0.94)	1.500	0.472
	Grayscale inverted	313 (98.43)	5 (1.57)		
	Std + Gsi	314 (98.74)	4 (1.26)		
Mass	Standard	293 (92.14)	25 (7.86)	3.111	0.211
	Grayscale inverted	299 (94.03)	19 (5.97)		
	Std + Gsi	297 (93.40)	21 (6.60)		
Axillary Lymph Node	Standard	248 (77.99)	70 (22.01)	2.375	0.305
	Grayscale inverted	246 (77.36)	72 (22.64)		
	Std + Gsi	243 (76.42)	75 (23.58)		

*Std + Gsi* standard plus grayscale inverted image

Among the three assessment methods, a significant difference was observed in the detection of macrocalcification. In std + gsi images more macrocalcification (93 images) were detected than in standard images (84 images) (Cochran’s Q = 8.375, *p* = 0.015). There was also a significant difference in microcalcification detection among the methods. Multiple comparisons between the methods showed that microcalcification was more often observed in grayscale inverted (53 images) and std + gsi images (81 images) than in standard images (31 images). Moreover, microcalcification was the most often observed in std + gsi images (81 images) (Cochran’s Q = 68.509, *p* < 0.001). Lastly, there was a significant difference in the detection of intramammary lymph nodes among the 3 assessment methods. In std + gsi images more intramammary lymph nodes (59 images) were detected than in standard images (49 images) (Cochran’s Q =6.461, *p* = 0.039).

## Discussion

In Turkey, yearly mammographic scanning beginning at 40 years of age is recommended [8]. The double-reading system is preferred as it decreases the false-negative rate in scanning mammograms. In previous studies, this system increased breast cancer detection rates by 4–15% [5, 9] and better detected small breast cancers owing to its relatively high sensitivity [10]. However, the double-reading system is impractical because it increases the cost of the mammogram and requires two readers. Moreover, it has been shown to decrease the positive estimation value and increase the recall rate and the patient’s anxiety level [11].

The CAD system is an alternative to the double-reading system, especially in busy centers [6]. The CAD system directs the radiologist’s attention to suspicious regions, thereby aiding detection of the lesion [12] Although the CAD system has existed for 10 years, its additive contribution to routine scanning is controversial [13]. In the study by Fiona et al., the double-reading system and CAD system had similar detection rates [6]. However, another study suggested that the CAD system improved diagnostic performance and reduced assessment time [14]. An additional benefit of the CAD system is its capacity to detect microcalcification even when used by radiologists with little experience [15, 16].

Mammographic sensitivity is lowest during the premenopausal period, at which time the fibroglandular tissue is especially dense. In this situation, digital mammography more efficiently detects lesions than does conventional mammography [17]. Digital mammography can also process the data in various formats because its image forming and display phases are different.

We assessed the usefulness of grayscale inverted images for detecting mammary abnormalities using digital mammography facilities when double reading or CAD system is not feasible. To our knowledge, there are previous studies in the medical literature that has performed such an assessment [18–20]. But we have not found a study on mammograms. Including grayscale inverted images in mammogram assessments is not routine; however, it is not a difficult application (standard images can be converted to grayscale inverted images by the touch of a button in digital systems). However, evaluation of the negative image, as well as the standard image, somewhat prolongs the assessment time.

Our study did not examine the distribution pattern, number, or morphological properties of microcalcifications except for their size. Therefore, were unable to categorize them as benign, suspicious, or malignant. An advantage of grayscale inverted images for detection of microcalcification is that black spots indicative of microcalcification are easily seen and taken attention on white backgrounds and graphs. By altering the perception of the reader, even a single white spot not apparent on a standard image becomes readily recognizable as a black spot on a negative image.

Additionally, intramammary lymph nodes were also easier to identify on grayscale inverted images than on standard images, which increases diagnostic and detection rates. We could readily detect the characteristic small fatty hilum of intramammary lymph nodes on negative images. However, grayscale inverted images provided no advantage in terms of detecting axillary lymph nodes, even though they also have a fatty hilum. In our opinion, this may be explained by the fact that the axillary lymph node hilum is large and therefore apparent on both negative and standard images. Evaluation of grayscale inverted images in addition to standard images aids diagnosis owing to the ease of detecting the small white fatty hila of intramammary lymph nodes, which appear as bean-shaped, darkly colored masses.

The limitations of our study are the small number of cases and the performance of the assessments by a single radiologist.

In our study, assessment of grayscale inverted images, compared with standard images, improved detection of microcalcifications and intramammary lymph nodes. We believe that this finding is important because microcalcification is a strong indicator of benign or malignant lesions. Hence, we recommend the use of grayscale inverted images for evaluating microcalcification.

## Conclusions

Assessment of grayscale inverted images in addition to standard images improves mammographic evaluation and facilitates the detection of small but crucial signs of breast cancer such as microcalcification. Therefore, assessment of grayscale inverted images can be practicable especially in busy centers.

## Abbreviations

ACR: American College of Radiology
BIRADS: Breast Imaging Reporting and Data System
CAD: Computer-aided detection
Gis: Grayscale inverted
Std + Gsi: Standard plus grayscale inverted image
Std: Standard

## References

[1] Miller AB (1992). Canadian National Breast Screening Study: I and II. Can Med Assoc J.

[2] Frisell J (1991). Randomized study of mammography screening-preliminary report on mortality in the Stockholm trial. Breast Cancer Res Treat.

[3] Fletcher SW (2003). Mammographic screening for breast cancer. N Engl J Med.

[4] Birdwell RL (2005). Computer-aided detection with screening mammography in a university hospital setting. Radiology.

[5] Thurfjell EL (1994). Benefit of independent double reading in a population-based mammography screening program. Radiology.

[6] Gilbert FJ (2008). Single reading with computer-aided detection for screening mammography. N Engl J Med.

[7] D’Orsi CJ (2013). ACR BI-RADS_®_ Atlas, Breast Imaging Reporting and Data System.

[8] Turkish Radiology Assosciation. Guide for Breast Cancer Scanning in Turkey. TRD Sufficiency Board; 2011.

[9] Hofvind S (2009). Screening-detected breast cancers: discordant independent double reading in a population-based screening program. Radiology.

[10] Blanks RG (1999). Observer variability in cancer detection during routine repeat (incident) mammographic screening in a study of two versus one view mammography. J Med Screen.

[11] Anderson ED (1994). The efficacy of double reading mammograms in breast screening. Clin Radiol.

[12] Azavedo E (2012). Is single reading with computer-aided detection (CAD) as good as double reading in mammography screening? A systematic review. BMC Med Imaging.

[13] Guerriero C (2011). Is computer aided detection (CAD) cost effective in screening mammography? A model based on the CADET II study. BMC Health Serv Res.

[14] Jung NY (2014). Who could benefit the most from using a computer-aided detection system in full-field digital mammography?. World J Surg Oncol.

[15] Bolivar AV (2010). Computer-aided detection system applied to full-field digital mammograms. Acta Radiol.

[16] Dromain C (2013). Computed-aided diagnosis (CAD) in the detection of breast cancer. Eur J Radiol.

[17] Pisano ED (2005). American College of Radiology Imaging Network digital mammographic imaging screening trial: objectives and methodology. Radiology.

[18] Park JB (2015). Diagnostic accuracy of the inverted grayscale rib series for detection of rib fracture in minor chest trauma. Am J Emerg Med.

[19] Thompson JD, et al. The impact of greyscale inversion for nodule detection in an anthropomorphic chest phantom: a free-response observer study. Br J Radiol. 2016;89:(1064):20160249. 10.1259/bjr.20160249 PMC5124894 27266374

[20] Reese DJ (2016). Intra- and interobserver variability of board-certified veterinary radiologists and veterinary general practitioners for pulmonary nodule detection in standard and inverted display mode images of digital thoracic radiographs of dogs. J Am Vet Med Assoc.

